# DRA-UNet for Coal Mining Ground Surface Crack Delineation with UAV High-Resolution Images

**DOI:** 10.3390/s24175760

**Published:** 2024-09-04

**Authors:** Wei Wang, Weibing Du, Xiangyang Song, Sushe Chen, Haifeng Zhou, Hebing Zhang, Youfeng Zou, Junlin Zhu, Chaoying Cheng

**Affiliations:** 1 Shendong Coal Branch, China Shenhua Energy Co., Ltd., Yulin 719000, China; hearttearsyeye19@163.com (W.W.); chensushe@sina.com (S.C.); 10021932@ceic.com (H.Z.); 2 School of Surveying and Land Information Engineering, Henan Polytechnic University, Jiaozuo 454000, China; jzitzhb@hpu.edu.cn (H.Z.); zouyf@hpu.edu.cn (Y.Z.); 212304020099@home.hpu.edu.cn (J.Z.); chengchaoying@home.hpu.edu.cn (C.C.)

**Keywords:** coal mining ground surface, crack delineation, deep learning, DAM, ASPP, DRA-UNet

## Abstract

Coal mining in the Loess Plateau can very easily generate ground cracks, and these cracks can immediately result in ventilation trouble under the mine shaft, runoff disturbance, and vegetation destruction. Advanced UAV (Unmanned Aerial Vehicle) high-resolution mapping and DL (Deep Learning) are introduced as the key methods to quickly delineate coal mining ground surface cracks for disaster prevention. Firstly, the dataset named the Ground Cracks of Coal Mining Area Unmanned Aerial Vehicle (GCCMA-UAV) is built, with a ground resolution of 3 cm, which is suitable to make a 1:500 thematic map of the ground crack. This GCCMA-UAV dataset includes 6280 images of ground cracks, and the size of the imagery is 256 × 256 pixels. Secondly, the DRA-UNet model is built effectively for coal mining ground surface crack delineation. This DRA-UNet model is an improved UNet DL model, which mainly includes the DAM (Dual Dttention Dechanism) module, the RN (residual network) module, and the ASPP (Atrous Spatial Pyramid Pooling) module. The DRA-UNet model shows the highest recall rate of 77.29% when the DRA-UNet was compared with other similar DL models, such as DeepLabV3+, SegNet, PSPNet, and so on. DRA-UNet also has other relatively reliable indicators; the precision rate is 84.92% and the F1 score is 78.87%. Finally, DRA-UNet is applied to delineate cracks on a DOM (Digital Orthophoto Map) of 3 km^2^ in the mining workface area, with a ground resolution of 3 cm. There were 4903 cracks that were delineated from the DOM in the Huojitu Coal Mine Shaft. This DRA-UNet model effectively improves the efficiency of crack delineation.

## 1. Introduction

Due to the relatively fragile overburden and shallow depth of underground mining, the ground surface cracks can be easily generated along with the mining activities [1]. These cracks can immediately result in ventilation trouble under the mine shaft, runoff disturbance, and vegetation destruction; therefore, they can threaten mining safety, groundwater, and vegetation health [2]. In the Loess Plateau, the loose structure and knotted growth characteristics of loess make the susceptible to destruction of the overburden. If the overburden destruction is too aggravated by underground mining, it may cause overburden collapse and lots of cracks appearing on the ground [3]. These coal mining ground surface cracks can play the role of ventilation channels to deliver oxygen from the ground surface air into the belowground coal mining area. The oxygen from the ground surface air continuously reacts with coal and coalbed methane, and this chemical interaction increases the temperature within the underground mining spaces. The coal naturally burns as the ambient temperature rises, resulting in a gas explosion, and more importantly, poses a significant threat to the safety of mining operations [4]. Additionally, these ground surface cracks directly damage buildings, transportation lines, and other geotechnical facilities and lead to mechanical destruction of plant roots, vegetation, and soil degradation, as well as ecological environment problems, such as soil and water loss [5]. As a consequence, the quick, accurate, and effective delineation of ground surface cracks becomes the crucial issue for underground mining disaster prevention and control. Furthermore, the quick delineation of the ground surface can promote ecological restoration for the green mine.

Currently, monitoring ground surface cracks in mining areas primarily relies on in situ observations. Information about these cracks can be obtained using total station theodolite or Global Positioning Systems (GPS). These methods are time consuming and laborious, and the results are often discontinuous and in the form of limited datasets. In addition, some surveys have shown that ground surface crack widths are often at the centimeter level [6,7], with a small number exceeding the sub-meter scale. Despite the sub-meter resolution capability of current optical satellite images, detecting all cracks remains a challenge, particularly for those with narrower widths. UAV photogrammetry technology, known for its exceptional adaptability to various terrains, swift response capabilities, and cost effectiveness, efficiently captures high-resolution images with centimeter-level precision on a regular and ongoing basis. This capability facilitates the dynamic monitoring of ground cracks within mining areas [8].

Ground surface crack delineation from UAV images involves imagery processing techniques [9]. Currently, a variety of technical methods are employed for crack processing in image analysis [10]. These approaches encompass traditional manual interpretation, where experts visually identify and label cracks. However, this method is susceptible to misclassification and omission due to the labor-intensive nature and inefficiency of operators manually delineating cracks based on their own expertise and experience [11]. The threshold segmentation method divides an image into different regions or objects by setting one or more threshold values. It is sensitive to noise, image quality, and variations in lighting, potentially affecting the choice of thresholds and the accuracy of segmentation. Additionally, for objects with irregular shapes or complex boundaries, threshold segmentation may not yield precise results [10]. The Canny algorithm is a widely used edge detection technique in the field of computer vision and image processing. Dual-threshold detection provides superior localization capabilities for weak edges, and hysteresis thresholding effectively detects and connects edges, maintaining high accuracy even in the presence of noise, yet it has poor generalization performance [12]. Machine learning techniques relying on feature engineering have been widely used in crack delineation. These techniques are more accurate than the traditional imagery processing methods but rely heavily on the characterization of manually extracted features [13]. Therefore, to accommodate a variety of unique conditions, innovative technologies and methods are necessary for the delineation of crack features in coal mining areas.

Deep learning is widely applied in the field of image processing. As a specific architecture in deep learning, the convolutional neural networks (CNNs) have outstanding advantages in automatically and effectively extracting image features, especially in the recognition and image crack delineation, where this efficient semantic segmentation method of CNNs shows significant advantages. For example, the CrackPix model [14] based on the Full Convolutional Network (FCN) can detect cracks automatically in concrete infrastructure without an artificial feature design, which is significantly superior to the traditional methods. Fan et al. [15] proposed a neural network U-HDN based on a codec structure, which integrates the crack context information into a multi-extension module to extract more crack features and improve crack detection performance. However, traditional convolutional neural network technologies usually rely on the feature input at a single scale, while ground cracks are long and narrow small-scale objects. Therefore, these networks need multi-scale feature information to achieve more accurate crack delineation [16]. To solve this problem, feature pyramids and hierarchical promotion networks (FPHBNs) [17] accurately detect cracks by integrating contextual information into low-level features. Chen et al. [18] enhanced the multi-scale features and performance of deep learning networks by using convolution and attention mechanisms with different expansion rates and proposed a method combining U-Net and the Multi-scale Global Inference module (MGRB) to significantly improve the efficiency and accuracy of ground crack detection.

The studies mentioned above are highly inspiring for our research. The presence of various types of grass, fallen leaves, and gravel, and the influence of varying lighting conditions and shadows on the coal mine site, make it challenging to accurately detect and extract cracks from the complex background of the surface crack images captured by the UAV. In addition, the lack of datasets for coal mine surface cracks is another issue that needs to be emphasized. Existing public datasets are mainly used for concrete surfaces, including pavements, rock cracks, architecture cracks, and brick wall cracks, such as Crack 500 [19] and DeepCrack [20], which are very different from the coal mining ground surface cracks because of cracks in the Loess Plateau are easily affected by topography and vegetation coverage.

To address these issues, an improved U-Net model is proposed in this paper. The RN module, DAM module, and ASPP module are introduced to construct the DRA-UNet model for more precise automatic delineation of surface cracks from UAV high-resolution photogrammetric images. The analysis demonstrates that integrating the RN module, DAM module, and ASPP module significantly enhances the ability to capture multi-scale features. This DRA-UNet model also improves the attention of cracks in loess areas, reduces interference from irrelevant information, and enhances the generalization capability of multi-level spatial and spectral features.

## 2. Materials and Methods

### 2.1. Study Area and Dataset Description

#### 2.1.1. Study Area

**Figure 1:**
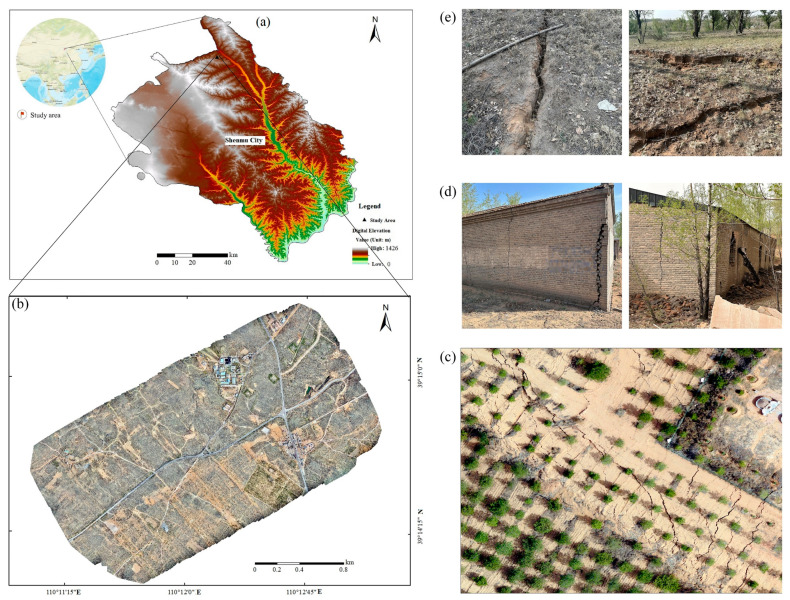
Overview of the study area: (**a**) location of the study area, (**b**) UAV mosaiced imagery, (**c**) imagery of some of the ground surface cracks captured by the UAV, (**d**) damage to the building from the ground surface crack, and (**e**) imagery of a ground crack.

The Huojitu Coal Mine Shaft of Daliuta Coal Mine (Figure 1) belongs to the Shenhua Shendong Coal Group and is situated at the border of Shaanxi Province (subordinate to the Daliuta Pilot Area of Shenmu City, Shaanxi Province) and Inner Mongolia Autonomous Region. The Huojitu Coal Mine Shaft of the Daliuta Coal Mine is bounded by the Wulanmulun River to the northeast. The Huojitu Coal Mine Shaft field covers an area of 63 km^2^. The topography and geomorphology of the area show a general pattern of higher elevation in the west and lower elevation in the east. The highest point in the area reaches an elevation of 1274 m, while the lowest point is at 1063 m. The average elevation is approximately 1203 m, and there is a maximum height difference of 211 m between points.

The research area is located at the border zone between the Loess Plateau and the Maowusu Desert in northern Shaanxi Province, which is a desert landform with aeolian deposits and is dominated by loess beams and desert mudflats and denuded hills. The nearby terrain is relatively flat and level, and the overall topography is high in the middle and low on the east and west sides in terms of topographic elevation. It has a typical mid-temperate semi-arid continental climate with sparse vegetation, and it is an ecologically fragile area. As a result of large-scale coal seam mining, land degradation has been aggravated, resulting in a series of problems around the mining area, particularly in geological and environmental terms, such as ground subsidence and surface cracks.

#### 2.1.2. GCCMA-UAV Dataset

**Figure 2:**
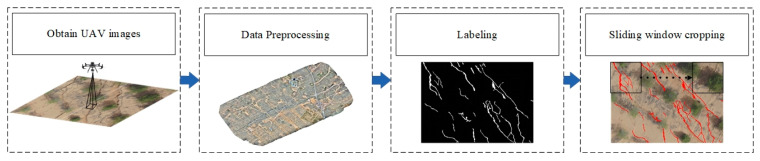
Construction of the ground crack dataset in the coal mining area.

The construction process of the Ground Cracks of the Coal Mining Area Unmanned Aerial Vehicle (GCCMA-UAV) dataset of UAV high-resolution images is shown in Figure 2. We utilized the high-resolution imagery data obtained by the UAV under clear weather conditions (for UAV-related parameter information, see Table 1) as the sample source for ground crack delineation. Subsequently, the raw data were processed using imagery mosaic software and generated orthographic images of the mining area. The UAV images were semantically labeled using visual interpretation, and the labeled images were divided into background cracks with a pixel value of 0 and ground cracks with a pixel value of 255. Due to the limitation of computational resources, we divided the images into 256 × 256 pixel-size blocks with an overlap rate of 25% by using a cropping method, resulting in 6280 ground crack samples. These samples were divided into training, validation, and test sets at a ratio of 8:1:1, and the dataset was named the surface crack dataset from UAV high-resolution imagery.

### 2.2. DRA-UNet Model for Surface Crack Delineation

The DRA-UNet model is specifically designed for the task of delineating ground surface cracks in coal mining areas using high-resolution UAV imagery. This section describes the model architecture in detail, highlighting the integration of key components and their roles in enhancing the model’s performance.

#### 2.2.1. Overall Structure

**Figure 3:**
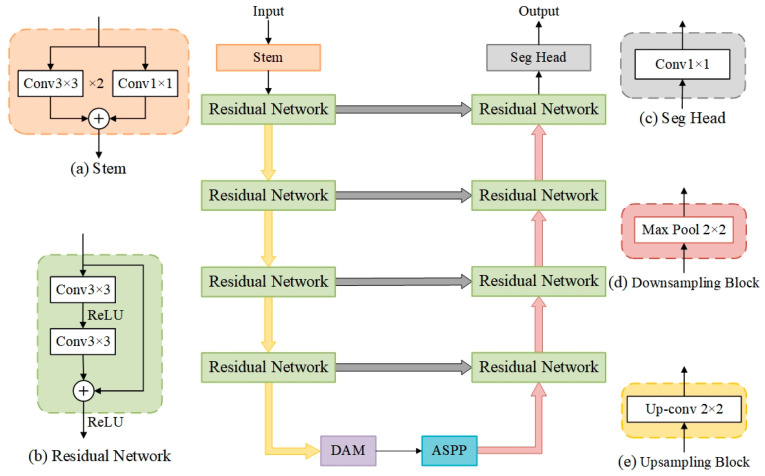
A schematic diagram of the DRA-UNet model.

The ground surface crack delineation DRA-UNet model mainly contains a DAM (Dual Attention Mechanism), RN (Residual Network), and ASPP (Atrous Spatial Pyramid Pooling), as shown in Figure 3. The residual module replaces the convolutional layer in the original U-Net network encoder and decoder [21], which enhances feature characterization and increases the depth of the network, allowing for more complex and layered feature extraction. At the bridge, the combination of the dual attention mechanism and the ASPP module is used in the model. The dual attention mechanism enhances the model’s attention to important features, while the ASPP captures contextual information at multiple scales. The Dual Attention Mechanism (DAM) is employed to assimilate contextual information within images, thereby facilitating the generation of higher-level features. The Atrous Spatial Pyramid Pooling (ASPP) module is tasked with extracting multi-scale information from the advanced features produced by the DAM. This effectively enhances the accuracy and robustness of surface crack delineation.

#### 2.2.2. The Residual Network (RN) Module

The RN is a deep convolutional neural network architecture that addresses the problem of gradient vanishing and model degradation in deep networks by introducing residual connections [22]. In traditional deep networks, the gradient becomes progressively smaller with the network layer increases, resulting in a network that is difficult to train. The increase in network layers also tends to lead to model degradation, i.e., an increase in the depth of the network reduces the model performance instead. To solve these problems, ResNet proposes the concept of residual connections. A residual connection is a kind of jump connection that adds the input directly to the output, and this connection is enabled so that the network can learn the residual information directly. 

The basic unit of the ResNet is the residual block, which comprises two convolutional layers. At each residual block, a residual connection is introduced between the input and the output, adding the input directly to the output. In this manner, the network can gradually adjust the output by learning the residuals rather than directly performing the entire mapping. This design allows the network to learn constant mappings more easily, thus facilitating the optimization of the network. By stacking multiple residual blocks, a deep ResNet can be constructed. The residual connection is a skipped connection, and it smoothens the gradients inside the network, hence preventing gradient vanishing. Additionally, the residual connection can alleviate the degradation of the model.

**Figure 4:**
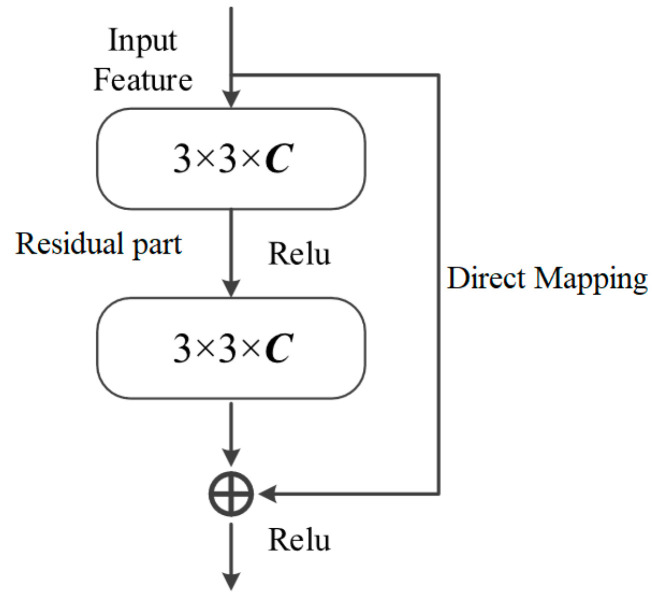
Structure of the residual network module.

As the network becomes deeper, training it becomes increasingly challenging. Therefore, to prevent network degradation, integrating shortcut connections in U-Net networks is especially crucial. In addition to this, to achieve more precise pixel-level predictions, the computationally intensive original convolutional layers are replaced with a Basic Block, as shown in Figure 4. 

Use an input feature map after two convolutions and ReLu functions, the resultant features are summed with the original input features to obtain the final output feature map [23]. Equation (1) is the representation of the residual block.

(1) $$x_{l+1}=x_{l}+f\left(x_{l},w_{l}\right)$$

**Figure 5:**
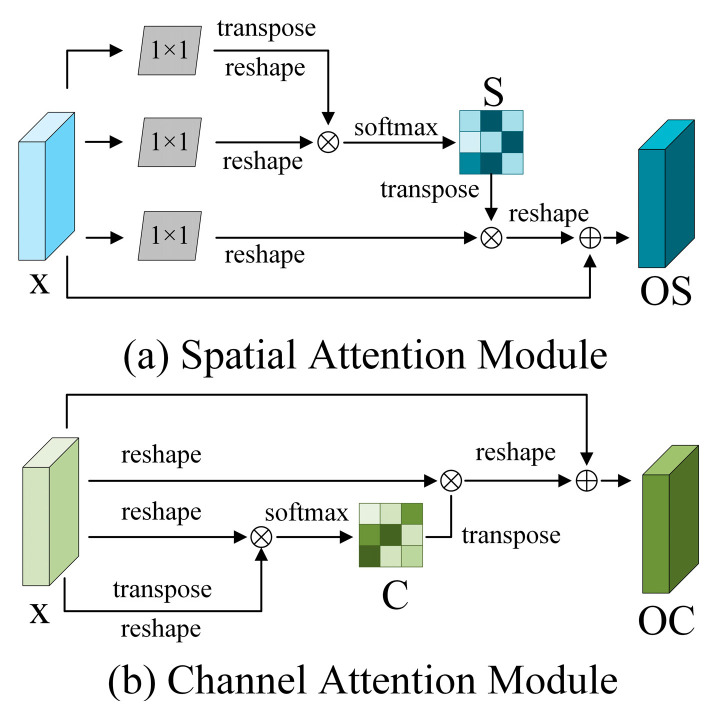
Structure of the DAM module.

The residual block includes a direct mapping part and a residual part. The direct mapping’s response corresponds to the polyline at 90° on the right side of Figure 5. The residual part is comprised of two convolution operations, and it is shown in the middle of Figure 4 as the 3 × 3 × C (channel) convolved part. The shortcut connection between the input and output feature maps transfers the crack information delineated from the former layer of the network to the latter layer. By avoiding information loss to a greater extent, this action effectively prevents network degradation resulting from the number of neural network layer increases.

#### 2.2.3. The Dual Attention Mechanism (DAM) Module 

The residual connection in the RN usually transmits information between adjacent layers, causing each layer to be limited to local features. Although this local capture efficiency is very high, the RN has difficulty integrating large amounts of global feature and context information. To enhance the U-Net network and capture global contextual information from the features extracted by the RN, we have introduced the DAM model [24]. In Figure 5, the DAM module consists of two parts: the SAM and CAM. The SAM has the ability to fuse with spatial context information, which can enhance the performance of identifying and describing the crack representation of crack pixel locations. Figure 5a illustrates the process of feeding the features into two conv 1 × 1 layers. This is followed by feature matrix transposition, reshaping, and multiplication. Then, output the results to generate the weighted spatial attention through the Softmax layer matrix. This matrix represents the spatial location connection between any two pixels. The effect of the i’th pixel on the j’th pixel can be expressed as $S_{i,j}$, as shown in Equation (2).

(2) $$S_{i,j}=\frac{\exp\left({\left(W_{1\times 1}x_{j}\right)}^{T}W_{1\times 1}x_{i}\right)}{\sum_{i=1}^{N}\exp\left({\left(W_{1\times 1}x_{j}\right)}^{T}W_{1\times 1}x_{i}\right)}$$

where *W*_1×1_ stands for the conv 1 × 1.

Then, the input feature *x* is again subjected to a conv 1 × 1 transform, and the fused feature is generated by multiplying spatial attention matrix *S* with the transformed feature. The final step is to perform an element-wise summation operation on the input feature x and the fused feature to generate the final feature map *OS*. *OS* is able to concentrate on specific contextual information related to cracks in an image, instead of simply considering all information. Therefore, *OS* is enabled to capture the data that are most instrumental for crack feature identification, which makes the extraction of crack features both efficient and effective. Meanwhile, the description of the model to identify cracks can remain stable and reliable under different environments or conditions. The fusion result $OS_{i}$ for the ith pixel is determined by Equation (3).

(3) $$OS_{i}=\alpha \sum_{j=1}^{N}S_{i,j}^{T}W_{1\times 1}x_{i}+x_{i}$$

*α* is initialized to 0, and gradually learns to allocate more weight.

The CAM can be used to capture dependencies between any two channels, identify the common effects of the corresponding features or semantic responses of different channels, reveal the complementary relationships between different levels of features, and more intuitively show the most important features in decision making, which are used to improve the performance and interpretability of the model. As shown in Figure 5b, matrix transposition, reformation, and multiplication operations are performed directly on the features, and then the Softmax layer yields a weighted channel attention matrix C, which models the interdependencies between any two feature map channels. In a feature map, $C_{i,j}$ or the degree of influence of channel j on channel i can be expressed as Equation (4).

(4) $$C_{i,j}=\frac{\exp\left(x_{j}^{T}x_{i}\right)}{\sum_{i=1}^{N}\exp\left(x_{j}^{T}x_{i}\right)}$$

The channel matrix *C* is multiplied with the input features to obtain the fusion features. The input features and the fusion features are then subjected to the elemental addition operation, the final output feature map *OC*. This process heightens the interconnection of information between channels, and the information from different channels can be more effectively integrated, helping to more accurately identify and classify fracture features. The resulting feature fusion $OC_{i}$ for the ith channel is calculated as Equation (5).

(5) $$OC_{i}=\beta \sum_{j=1}^{N}\left(C_{i,j}x_{i}\right)+x_{i}$$

where *β* gradually learns weights from 0. Finally, the high-level semantic features of the SAM and CAM outputs are obtained by integrating the global spatial position and channel features.

#### 2.2.4. The Atrous Spatial Pyramid Pooling (ASPP) Module

The ASPP module is a widely utilized technique in semantic segmentation tasks and is designed to leverage convolution operations with varying expansion rates for capturing multi-scale information and enhancing the model’s contextual understanding [25]. Traditional ASPP architectures typically feature multiple parallel branches, each applying distinct expansion rates to enlarge the receptive field of the convolution kernel without significantly increasing computational complexity. Standard ASPP modules commonly include a 1 × 1 convolution branch and several 3 × 3 extended convolution branches with progressively increasing expansion rates to capture image features at multiple scales. Additionally, to compensate for the limited ability of convolution operations to capture global context information, a global average pooling branch is often incorporated.

**Figure 6:**
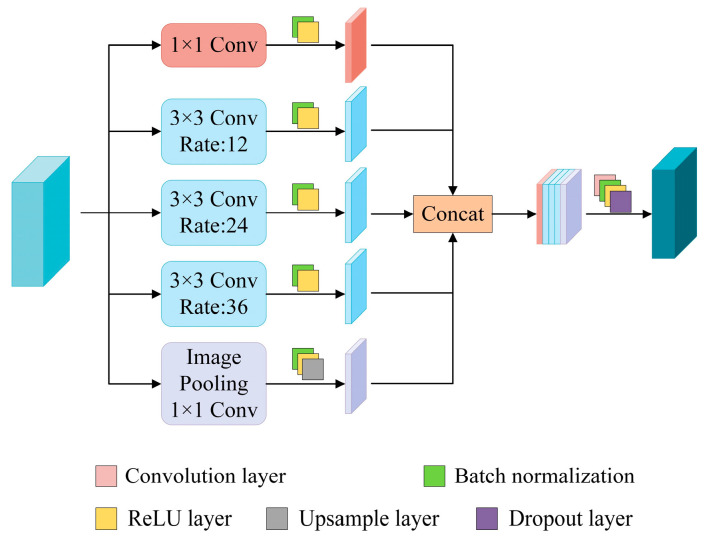
Structure of the ASPP module.

In our approach, we have optimized the traditional ASPP structure to address strong noise interference caused by complex backgrounds in drone images. Figure 6 shows the structure of the ASPP module. Specifically, we process feature maps through five parallel branches, comprising a 1 × 1 convolution layer branch, three 3 × 3 convolution layer branches with expansion rates of 12, 24, and 36 respectively, and a branch that combines image pooling and a 1 × 1 convolution layer. It is important to note that as the expansion rate approaches the size of the feature map, the effectiveness of the 3 × 3 convolution kernel may diminish into that of a 1 × 1 convolution kernel, which weakens its ability to capture global context information. To resolve this issue, we introduce global average pooling operation and incorporate batch normalization (BN) as well as modified linear unit (ReLU) activation functions after each convolutional branch to prevent gradient disappearance and expedite model convergence.

Finally, during the output phase of the ASPP module, we merge feature maps generated by each branch to integrate results from multi-scale resampling. This enhanced ASPP module not only fortifies multi-scale information capture but also enhances classification accuracy across different scale regions, thereby rendering our optimized U-Net network more precise and efficient in detecting cracks within UAV images featuring complex backgrounds.

## 3. Results

### 3.1. Experimental Setup

This experiment is implemented based on the PyTorch framework. The CPU used for training is an Intel(R) Xeon(R) W-2265 CPU @ 3.50 GHz and the GPU is a NVIDIA RTX 4000 with a running memory of 128 G on a 64-bit Windows operating system. PyTorch version 2.0.0 with an initial learning rate set to 5 × 10^−4^, a training period set to 100, a batch size set to 16, and an optimizer set to Adam was used.

### 3.2. Data Enhancement

In the experiments, a data augmentation technique is employed to enhance the robustness of the model, mitigate overfitting, and at the same time improve the generalization ability of the model, and this data enhancement operation is only used for training sets. Three different types of data enhancement were implemented as follows:

(1) Random adjustment of brightness and contrast;
(2) Flip horizontally and vertically randomly;
(3) Randomly rotate 90 degrees, 180 degrees, and 270 degrees.

### 3.3. Loss Function

When segmenting ground surface cracks in mining areas, it is essential to consider both segmentation precision and recall rates. The Dice Loss presented in Equation (6) offers a more precise evaluation of the correspondence between the predicted results and actual values [26]. This prioritizes coverage of cracked regions during training sessions, leading to improved segmentation recall rates. Nevertheless, this type of loss solely emphasizes similarity without penalizing misclassifications, thereby limiting its effectiveness at enhancing model accuracy.

Equation (7)’s binary cross-entropy (BCE) loss primarily serves binary classification tasks by computing differences between the predicted results and actual values while effectively penalizing false classifications [27]. However, within ground surface crack segmentation tasks where cracked regions constitute a smaller proportion within overall imagery contextually leads models towards predicting these regions as background elements instead.

To mitigate class imbalance effects within such scenarios, class weights are introduced into adjusting our loss functions; controlling contributions from positive samples through weight adjustments allows models greater focus on capturing crucial cracked information.

DRA-UNet strategically combines both Dice Loss with BCE losses, harnessing their respective strengths and resulting in enhanced overall model performance as depicted by their combined representation shown in Equation (8).

(6) $$L_{Dice}=1-\frac{2\sum_{i=1}^{N}p_{i}q_{i}+\varepsilon }{\sum_{i=1}^{N}p_{i}+\sum_{i=1}^{N}q_{i}+\varepsilon }$$

(7) $$L_{BCE}\left(p_{i},q_{i}\right)=-\frac{1}{N}\sum_{i=1}^{N}\left[p_{i}\log\left(q_{i}\right)+\left(1-p_{i}\right)\log\left(1-q_{i}\right)\right]$$

(8) $$L_{BD}\left(p_{i},q_{i}\right)=\alpha L_{BCE}+\beta L_{Dice}$$

where *N* is the number of pixels, *qi* is the actual category, *pi* is the predicted category, and *ε* is the smoothness coefficient.

### 3.4. Comparative Methods

To prove the accuracy and feasibility of the DRA-UNet model, we compared the evaluation indicators of DeepLabV3+, SegNet, PSPNet, Segformer, and FastSCNN with the DRA-UNet model as follows.

(1) DeepLabV3+

DeepLabV3+ is the depth of the image semantic segmentation for a neural network [28]; it combines Atrous convolution and spatial pyramid pool (spatial pyramid pooling, ASPP) while maintaining a high calculation efficiency, and it enhances the ability of the object model to capture different dimensions. DeepLabV3+ is one of the important models in semantic segmentation and can capture features at multiple scales over a wide perception field.

(2) SegNet

SegNet is an end-to-end deep learning network [29]. Through the input imagery, features are extracted through the encoder network, and the spatial resolution of the image is restored by the decoder network. Finally, the category of each pixel is exported through the Softmax classifier to realize the semantic segmentation of the imagery.

(3) PSPNet

PSPNet (Pyramid Scene Parsing Network) introduces the Pyramid Pooling Module, which captures global and local contextual information through pooling operations at different scales to improve the performance of semantic segmentation [30]. PSPNet has excellent performance in dealing with complex scenes and multi-scale objects. PSPNet performs well in handling complex scenes and multi-scale objects and is widely used in tasks such as cityscape segmentation.

(4) Segformer

Segformer is a semantic segmentation model that combines the advantages of the transformer architecture and convolutional neural networks (CNNs) [31]. By using an efficient transformer encoder to extract global features, combined with a lightweight decoder for fine-grained segmentation, Segformer achieves a good balance between accuracy and computational efficiency and is suitable for a wide range of semantic segmentation application scenarios.

(5) FastSCNN

FastSCNN is an efficient semantic segmentation model designed for mobile devices and embedded systems [32]. It adopts a lightweight architecture, including a learning imagery downsampling module, a global feature extraction module, and a feature fusion module, which can significantly reduce the computational overhead and memory usage while ensuring segmentation accuracy, making it well suited for real-time applications.

### 3.5. Evaluation Indicators

In our experiments assessing the model’s efficacy and accuracy, we utilized key performance metrics, such as the Pr (precision rate), Re (recall rate), F1 (F1 score), and MIoU (mean intersection over union), which are defined below.

(9) $$Pr=\frac{TP}{TP+FP}$$

(10) $$Re=\frac{TP}{TP+FN}$$

(11) $$F1=\frac{2\times Pr\times Re}{Pr+Re}$$

(12) $$MIoU=\frac{1}{2}\left(\frac{TP}{TP+FP+FN}+\frac{TN}{TN+FN+FP}\right)$$

In imagery segmentation tasks, TPs, FPs, FNs, and TNs, respectively, correspond to the true positives, false positives, false negatives, and true negatives in the confusion matrix, as shown in Table 2. When dealing with highly unbalanced samples, the key is to reduce false negatives (FNs) to ensure that as many cracks as possible are correctly identified. Therefore, for crack segmentation tasks, recall is more significant than precision. Moreover, it is important to note that precision or recall needs to be traded off in the experiment. High-sensitivity models usually lead to higher recall rates but may have less precision. A lower sensitivity model may improve precision, but the recall rate will decrease. To balance the two, F1 scores were introduced. The F1 score is the harmonic average of precision and recall. When both precision and recall are higher, the F1 score will also be higher, which is suitable for cases where both accuracy and recall need to be optimized. In addition, MIoU (mean intersection over union) is a measure of the similarity between actual cracked pixels and predicted cracked pixels. Therefore, the higher the value of MIoU, the higher the similarity between the model-predicted cracks and the real cracks, reflecting the better segmentation effect.

### 3.6. Experiments

On the GCCMA-UAV dataset, we conducted comparison experiments using DRA-UNet and five other comparison methods: DeepLabV3+, SegNet, PSPNet, Segformer, and FastSCNN’s semantic segmentation method.

**Figure 7:**
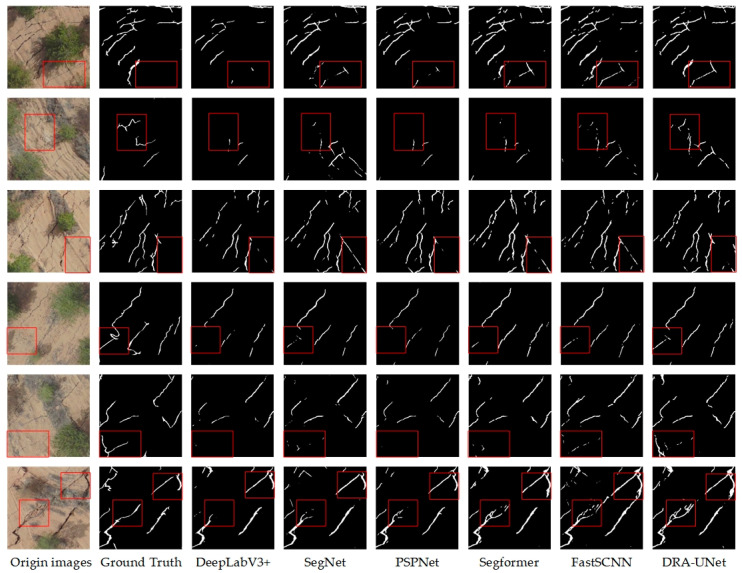
Crack delineation by different models using the GCCMA-UAV dataset.

The crack delineation results for all six methods are shown in Figure 7**.** Every DL method yields superior recognition outcomes for cracks that are visible to the naked eye. However, upon closer examination, it is evident that the delineation results of DRA-UNet in the first row of red frames are more precise and closely resemble the ground truth of the original imagery, similar to the delineation results shown in the first three rows of red rectangles. In addition, DRA-UNet can accurately recognize cracks that are difficult to distinguish with the naked eye or that are not clearly defined enough, as seen in the final three rows in the red frame. In contrast, other DL methods cannot depict a clear and complete boundary of the real shape of ground cracks, which highlights the advantage of DRA-UNet in solving complex cases.

In the coal mining area, our network exhibits an obvious performance advantage on the ground crack dataset in Table 3. Because of the highest recall rate of 77.29% in DRA-UNet, our network shows phenomenal performance in capturing most ground cracks in effect, even though its precision is slightly lower than DeepLabV3+. MIoU demonstrates a significant level of 69.88%, whereas F1 also achieves 78.87%, demonstrating the superior performance of the model in segmentation quality and overall performance.

### 3.7. Ablation Experiments

Table 4 presents the results of the ablation experiments, comparing the performance of different models on four metrics. U-Net + RN. In contrast to the basic U-Net, upon the addition of the RN module, the Pr slightly declined (from 81.35% to 80.65%), while the Re increased (from 73.46% to 76.86%). Nevertheless, F1 remained unchanged, and MIoU also slightly decreased (from 67.90% to 66.90%). This might suggest that the incorporation of the RN enhanced the ability to capture details, thereby elevating the recall rate. However, due to the potential introduction of some redundant information, it resulted in a marginal decrease in precision. U-Net + DAM. After introducing the DAM module, the Pr significantly rose (from 81.35% to 84.76%), but the Re significantly dropped (from 73.46% to 71.41%), ultimately leading to a slight reduction in F1 (from 76.81% to 76.43%), and MIoU also decreased (from 67.90% to 65.57%). The attention mechanism of the DAM was conducive to improving the precise positioning of the target area, thereby enhancing the precision. Nevertheless, it might cause missed detections as it focused more on specific areas, thereby lowering the recall rate. U-Net + ASPP. After adding the ASPP module, the Pr slightly decreased (from 81.35% to 80.01%), but the Re significantly increased (from 73.46% to 76.63%), resulting in an increase in F1 to 78.21%. However, MIoU decreased to 65.25%. ASPP enhanced the model’s perception ability for targets of different sizes through multi-scale feature extraction, improving the recall rate. However, it might lead to a decrease in precision due to an increase in misclassification of the background area. U-Net + RN + DAM. After combining the RN module and the DAM module, the Pr decreased to 77.90%, the Re increased to 74.08%, F1 slightly improved to 75.84%, and MIoU remained at 66.96%. This combination balanced the precision and the recall rate. However, compared with the individual use of the RN or DAM, the effect was not as anticipated. This might be because the functions of these two modules had some redundancies or conflicts, leading to no significant improvement in performance. U-Net + RN + ASPP. After combining the RN and ASPP, the Pr further decreased to 78.50%, the Re slightly increased to 76.08%, F1 increased to 73.84%, and MIoU increased to 68.66%. This combination was beneficial for improving the overall target detection ability. However, the decrease in Pr might be attributed to the increase in model complexity, making it more challenging to accurately distinguish boundaries. U-Net + DAM + ASPP. After combining the DAM module and the ASPP module, the Pr increased to 82.54%, the recall slightly decreased to 73.29%, F1 was 75.87%, and MIoU was 65.68%. This indicated that the combination of the two could be complementary, but it might also give rise to some contradictions. For instance, the DAM tended to focus on important areas, while ASPP focused on multi-scale, which might lead to information conflicts. DRA-UNet. The final DRA-UNet incorporated multiple modules and performed optimally. The Pr reached 84.92%, the Re reached 77.29%, F1 was 78.87%, and MIoU also reached 69.88%. This outcome suggested that under a reasonable architectural design, the combination of different modules could be complementary and maximize performance. Among them, the DAM strengthened the attention mechanism, ASPP provided multi-scale information, and the RN assisted in stabilizing the gradient and optimizing the model training process, thereby enabling DRA-UNet to achieve superior results in all metrics. 

### 3.8. Model Generalization Study

In this section, we employ the publicly accessible Crack500 dataset to examine the generalization capabilities of the DRA-UNet model. The Crack500 dataset comprises 500 images of road cracks, each with a resolution of 2000 × 1500 pixels, captured using mobile phones. These 500 images have been cropped to generate a total of 3368 smaller images, each measuring 256 × 256 pixels. Among these, 2696 images are designated for the training set, while 336 images are allocated for both the test and verification sets.

**Figure 8:**
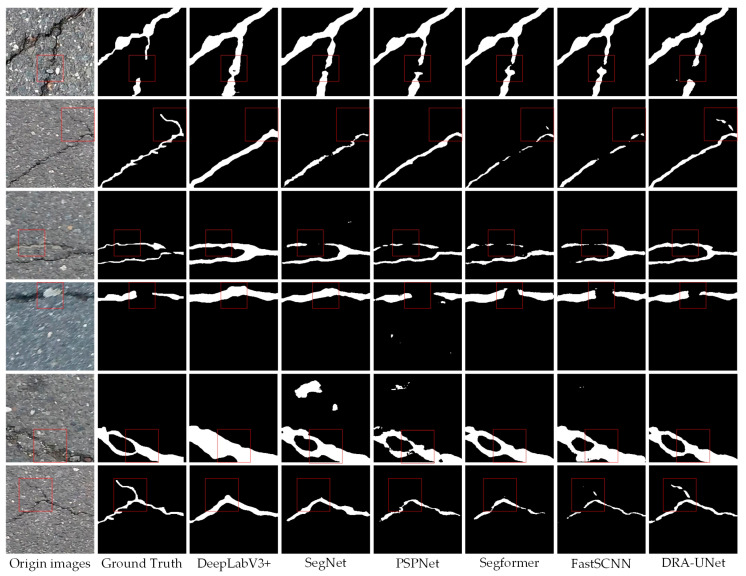
Crack delineation by different models using the Crack500 dataset.

Figure 8 presents a comparative analysis of the performance of the DR-UNet model against other models for crack identification within the Crack500 dataset. The findings indicate that, in contrast to alternative models, the DR-UNet model effectively and accurately detects subtle and ambiguous cracks while demonstrating superior noise suppression capabilities; consequently, its recognition results are more closely aligned with actual images. 

Table 5 presents a comparative analysis of the performance of the DRA-UNet model against other models on the Crack500 dataset. Within this dataset, the DRA-UNet model demonstrated exceptional performance, achieving the highest MIoU at 80.32%. While its precision was lower than that of Segformer and FastSCNN, it outperformed other models in terms of recall and F1 score. These results indicate that the DR-UNet model exhibits strong generalization capabilities and robustness when applied to the Crack500 dataset.

## 4. Discussion

Here, the comparison between DRA-UNet and other methods regarding crack delineation is shown in Table 6. A deep regression model named Faster R-CNN_YOLO was applied to delineate cracks in high-resolution images with dirty walls, pavements, marbles, etc., of different material types [33]. DMA-Net, including DeepLabv3+, ASPP, and the multi-attention module, was used for pavement crack detection. DMA-Net was applied to the Crack500 dataset, the DeepCrack dataset, and the Fma (Fitchburg Municipal Airport) dataset, and it showed excellent performance in road pavement crack segmentation, even in pavement crack images not captured with professional mapping equipment [34]. DDR-UNet (Deformable Dense Residual UNet) included the deformable convolution and deformable dense residual UNet. It was evaluated on three datasets (including an ore dataset) for ore imagery segmentation and measuring ore particle size distribution [35]. The GFSegNet (Ground Fissure Segmentation Network) included a DSDE (deep–shallow decoupled encoder), an MFFD (multi-scale feature fusion decoder), and loss function. The Mine Ground Fissure Unmanned Aerial Vehicle dataset (MGF-UAV) was used for crack delineation, and the spatial resolution of the dataset is 2.6 cm. The model was applied to the Crack500, DeepCrack, CrackForest, and ISPRS-Postdam datasets [36]. MFPA-Net mainly included the MFPN (Multi-scale Feature Pyramid Network) module, ASPP module, DRN (an improved Dilated Residual Network) module, and the DAM module, the the GFCMA (Ground Fissures of the Coal Mining Area) dataset was built to train the MFPA-Net model [37]. For MFPA-Net, the ground resolution of the crack imagery was 33 cm. MFPA-Net focused on the multi-scale spatial resolution features of the ground cracks. MFPA-Net paid more attention to the transmission of other resolution features of cracks to the delineation of coal mine ground surface cracks in mining areas. However, this DRA-UNet is inclined to fuse spatial and spectral features of the ground cracks from UAV images. When using MFPA-Net to process real large-scale scene images, the pixels on each image correspond to a real distance of 33 cm on the ground. Our GCCMA-UAV dataset has a ground resolution of 3 cm, and it can be used to delineate cracks in centimeter widths. Compared to the other five models, DRA-UNet shows a relatively same level of crack delineation (Table 6). The DRA-UNet model was applied well in the processing of crack delineation in coal mine ground surface area (Figure 7). This indicates that DRA-UNet pays more attention to the fusion of the spatial and spectral features of cracks to enhance its anti-noise capability.

**Figure 9:**
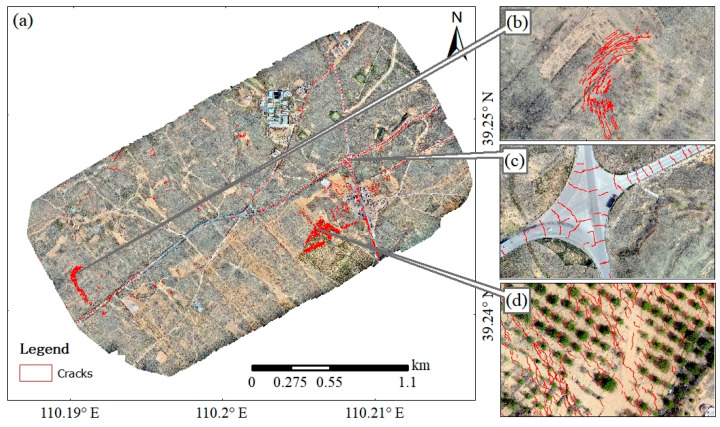
Crack delineation of the coal mining ground surface by UAV images: (**a**) cracks in the coal mining ground surface, (**b**) cracks in the mining area, (**c**) cracks in concrete pavement, (**d**) cracks in the woods.

This study mainly focuses on semantic-level segmentation of ground cracks. However, the size and shape of these ground cracks are also important because this vector information can be used to track changes in cracks, monitor ground movement and deformation, and provide data support for the prevention and response to potential disasters. Future research could explore ways to comprehensively capture and utilize vector information to improve monitoring accuracy. The DRA-UNet model was used to delineate cracks in the coal mining ground surface of the Huojitu Coal Mine Shaft (Figure 9a). There were 4903 cracks that appeared in the loose areas of the coal mining ground surface; the average length of these cracks was 5.82 m and the average width was 5.9 cm. Especially, the cracks easily appeared in the mining area (Figure 9b), concrete pavement (Figure 9c), and the woods (Figure 9d). In the mining area, mining-induced subsidence has led to the formation of annular crack groups. Due to the hardness of the concrete pavement, the cracks were difficult to heal. In the woods, the cracks were very short and small.

Ground surface cracking represents the primary manifestation of land degradation resulting from high-intensity coal mining. UAV photogrammetry provides a good solution for the rapid acquisition of ground cracks. This study presents a DL network framework, DRA-UNet, which is designed to address the challenges of ground surface crack detection in coal mine areas. The framework integrates the RN, DAM, and ASPP modules, which can automatically and accurately extract ground surface cracks in UAV images under complex backgrounds. In order to obtain ground surface crack information automatically, quickly, and accurately from UAV images and realize the development of DL technology in ground surface monitoring, in this study, the GCCMA-UAV dataset was constructed based on the UAV images collected in the coal mine area. In this study, experiments are performed on a GCCMA-UAV using various methods, and the experimental results show that fine crack segmentation can be achieved by DRA-UNet, which is significantly better than similar DL networks, particularly for fine and fuzzy cracks in complex backgrounds. 

The ablation research demonstrates that the combination of the RN, DAM, and ASPP modules enhances the performance of DRA-UNet greatly. DRA-UNet has shown remarkable reliability in comprehending global attention at a large scale and capturing intricate details at a micro-level. We have identified substantial issues with existing models that have difficulties in effectively representing the spatial structural links of cracks. As a result, these models often fail to maintain the continuity of cracks and tend to overlook smaller cracks. Additionally, other models often yield incorrect classifications due to noise factors, such as vegetation and shadows. The results of the experiments indicate that our network exhibits excellent reliability in ground crack segmentation. 

The method proposed in this paper is suitable for the rapid delineation of sparsely vegetated surface cracks in the Loess Plateau. The approach presented in this paper requires surface crack monitoring data, which are performed based on optical UAV images and which are sensitive to weather conditions and affected by factors such as vegetation cover. For regions with denser vegetation, combining multiple data sources should be considered to improve the reliability of monitoring, such as adding thermal infrared images. 

In this study, single-phase UAV images are used to identify surface cracks. However, the surface cracks will continue to appear over time around the actual coal mining area. Therefore, the change detection of surface cracks has become particularly important. Detecting changes in surface cracks using deep learning techniques is a very worthwhile research direction.

## 5. Conclusions

Building upon the generalization of the validation model utilizing a common dataset, the DR-UNet model introduced in this paper not only demonstrates exceptional crack recognition performance on specific datasets (such as the GCCMA-UAV dataset) but also exhibits commendable robustness and adaptability across various types of crack image data. The model’s generalization capability has been validated, confirming that DR-UNet can be effectively employed with other similar high-resolution UAV imagery datasets. This further underscores the model’s extensive applicability in practical scenarios. 

(1) Efficient deep learning model. DRA-UNet significantly enhances the feature extraction capability and crack identification accuracy by incorporating a residual network, dual attention mechanism, and void space pyramid pool module.

(2) Data augmentation strategy. Various data augmentation techniques are employed to enhance sample diversity, mitigate model overfitting, and improve the model’s generalization ability under complex backgrounds.

(3) Multi-scale feature extraction. The ASPP module effectively enhances fine crack extraction by capturing multi-scale features.

(4) Experimental validation. The results from experiments on the GCCMA-UAV dataset demonstrate that DRA-UNet outperforms existing similar models in metrics of Pr, Re, F1, and MIoU, showcasing its superiority and broad applicability for surface crack identification in coal mines.

Future research can further optimize the model structure and explore additional DL techniques to enhance the accuracy and efficiency of surface crack identification. Furthermore, constructing larger and more diverse datasets of surface fractures is also essential for enhancing the model’s performance. In summary, DRA-UNet offers an efficient and reliable solution for automatically identifying surface cracks in coal mines using high-resolution images.

## Figures and Tables

**Table 1:** UAV imagery data parameters.

Parameter Name	Performance Indicators
Type of drone	Vertical take-off and landing fixed wing
Band type	True color imaging
Imagery size	4056 × 3040 pixels
Date of flight	18 March 2023
Aerial heights	1190 m
Focus distance	40–60 mm
Exposure time	1/640 s
Ground resolution	3 cm

**Table 2:** Confusion matrix.

Confusion Matrix		Predicted Results	
		Cracks	Backgrounds
Ground Truth	Cracks	TP	FN
	Backgrounds	FP	TN

**Table 3:** Comparison of the evaluation metrics among the different models.

Model	Pr (%)	Re (%)	F1 (%)	MIoU (%)
DeepLabV3+	**85.44**	67.95	73.71	65.10
SegNet	76.55	68.46	70.81	63.59
PSPNet	84.76	71.41	76.43	67.57
Segformer	80.01	76.63	78.21	69.25
FastSCNN	77.90	74.08	75.84	66.96
DRA-UNet	84.92	**77.29**	**78.87**	**69.88**

The bold font indicates the optimal value.

**Table 4:** Results of the ablation experiments.

Model	Pr (%)	Re (%)	F1 (%)	MIoU (%)
U-Net	81.35	73.46	76.81	67.90
U-Net + RN	80.65	76.86	76.81	66.90
U-Net + DAM	84.76	71.41	76.43	65.57
U-Net + ASPP	80.01	76.63	78.21	65.25
U-Net + RN + DAM	77.90	74.08	75.84	66.96
U-Net + RN + ASPP	78.50	76.08	73.84	68.66
U-Net + DAM + ASPP	82.54	73.29	75.87	65.68
DRA-UNet	**84.92**	**77.29**	**78.87**	**69.88**

The bold font indicates the optimal value.

**Table 5:** Performance comparison of DRA-UNet and other models on the Crack500 dataset.

Model	Pr (%)	Re (%)	F1 (%)	MIoU (%)
DeepLabV3+	76.12	72.13	74.07	77.84
SegNet	73.28	75.96	73.28	76.64
PSPNet	76.55	77.89	77.21	80.01
Segformer	79.21	75.88	77.51	80.26
FastSCNN	**80.85**	72.49	76.44	79.54
DRA-UNet	78.94	**78.86**	**78.90**	**80.32**

The bold font indicates the optimal value.

**Table 6:** Comparison between DRA-UNet and other models on crack delineation.

Model	Pr (%)	Re (%)	F1 (%)	MIoU (%)
Faster R-CNN_YOLO [33]	78.30	67.20	72.30	/
DMA-Net [34]	86.90	87.10	87.00	/
DDR-Unet [35]	96.68	82.88	/	75.99
GFSegNet [36]	80.98	90.56	85.50	74.67
MFPA-Net [37]	69.40	70.70	70.00	75.10
Our DRA-UNet	84.92	77.29	78.87	69.88

## Data Availability

Data will be made available on request.
